# Modified FOLFIRINOX versus sequential chemotherapy (FOLFIRI/FOLFOX) as a second‐line treatment regimen for unresectable pancreatic cancer: A real‐world analysis

**DOI:** 10.1002/cam4.4512

**Published:** 2021-12-24

**Authors:** Shun Tezuka, Makoto Ueno, Ritsuko Oishi, Shuhei Nagashima, Yusuke Sano, Kuniyuki Kawano, Satoshi Tanaka, Taito Fukushima, Hiroyuki Asama, Naoki Konno, Satoshi Kobayashi, Manabu Morimoto, Shin Maeda

**Affiliations:** ^1^ Department of Hepatobiliary and Pancreatic Medical Oncology Kanagawa Cancer Center Yokohama Japan; ^2^ Department of Gastroenterology Yokohama City University Graduate School of Medicine Yokohama Japan

**Keywords:** FOLFIRI, FOLFIRINOX, FOLFOX, pancreatic cancer, second‐line treatment

## Abstract

**Background:**

Although second‐line treatment for pancreatic cancer has been proven to have survival benefit, it is not clear which is the most preferred regimen. This study compared the efficacy and safety of modified FOLFIRINOX (mFOLFIRINOX) and sequential chemotherapy (FOLFIRI/FOLFOX) as a second‐line treatment regimen for unresectable pancreatic cancer.

**Method:**

This was a retrospective single‐center analysis of all patients who initiated treatment with mFOLFIRINOX or sequential chemotherapy from December 2014 to May 2019 as a second‐line treatment for unresectable pancreatic cancer. The sequential chemotherapy group included all patients who initiated sequential chemotherapy. For efficacy analysis, the primary endpoint was overall survival (OS) of all patients, excluding those with locally advanced pancreatic cancer. For safety analysis, we assessed the incidence of grade ≥3 adverse events in all patients.

**Results:**

Seventy‐four patients **(**mFOLFIRINOX group, *n* = 44; sequential chemotherapy group, *n* = 30) were included. OS tended to be slightly prolonged in the mFOLFIRINOX group than in the sequential chemotherapy group (median 10.6 [95% confidence interval {CI} 5.9–13.8] vs. 8.5 [95% CI 5.0–12.2] months; hazard ratio 1.40 [95% CI 0.71–2.71]). The objective response rate and disease control rate were 8.1% and 64.9%, respectively, in the mFOLFIRINOX group and 3.8% and 42.3%, respectively, in the sequential chemotherapy group. In safety analysis, the grade ≥3 rates of neutropenia, febrile neutropenia, and anorexia were 40.9%, 6.8%, and 18.2%, respectively, in the mFOLFIRINOX group and 3.3%, 0%, and 3.3%, respectively, in the sequential chemotherapy group.

**Conclusions:**

Whereas efficacy tended to be slightly better in the mFOLFIRINOX group than in the sequential chemotherapy group, given the higher incidence of grade ≥3 adverse events with mFOLFIRINOX than with sequential chemotherapy, sequential chemotherapy is a regimen with better risk–benefit balance than mFOLFIRINOX, and can be considered a second‐line treatment option for patients with unresectable pancreatic cancer.

## Graphical abstract

We compared the efficacy and safety of mFOLFIRINOX and sequential chemotherapy for second‐line treatment of unresectable pancreatic cancer. In efficacy results, the Kaplan–Meier curves showed that OS, the primary endpoint, tended to be consistently higher in the mFOLFIRINOX group than in the sequential chemotherapy group, however, the difference was not significant.

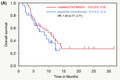

## 1 INTRODUCTION

Pancreatic cancer is the seventh leading cause of cancer deaths worldwide, with approximately 460,000 new cases and 430,000 deaths reported globally in 2018 and a 5‐year survival rate of <10%.^1^, ^2^ Most patients with pancreatic cancer (80%–90%) are diagnosed at an advanced stage^2^ and are not eligible for surgery because of vascular involvement or distant metastases. Therefore, systemic chemotherapy is recommended for patients with unresectable pancreatic cancer.^3^ When Eastern Cooperative Oncology Group (ECOG) performance status (PS)^4^ is good, the standard chemotherapy for unresectable pancreatic cancer is FOLFIRINOX (5‐fluorouracil [5‐FU], leucovorin [LV], irinotecan, and oxaliplatin),^5^ modified FOLFIRINOX (mFOLFIRINOX),^6^ or gemcitabine (GEM) plus nab‐paclitaxel (GnP).^7^ The increased survival provided by these standard first‐line chemotherapy regimens has enabled more patients to receive second‐line chemotherapy.^8^ Approximately 40% of patients with unresectable pancreatic cancer can receive second‐line chemotherapy.^9^

For patients previously treated with fluoropyrimidine‐based chemotherapy regimens such as FOLFIRINOX or mFOLFIRINOX, the use of GEM as monotherapy or as combination therapy is recommended as the subsequent chemotherapy.^3^, ^10^ Alternatively, for patients previously treated with GEM‐based chemotherapy, fluoropyrimidine‐based chemotherapy is recommended as the subsequent chemotherapy.^3^, ^10^ The National Comprehensive Cancer Network (NCCN) 2021 guidelines^11^ recommend liposomal irinotecan (nal‐IRI) plus 5‐FU/LV (NAPOLI‐1), FOLFIRI (5‐FU, LV, and irinotecan), FOLFIRINOX or mFOLFIRINOX, OFF (5‐FU, LV, and oxaliplatin), and FOLFOX (5‐FU, LV, and oxaliplatin) as second‐line fluoropyrimidine‐based chemotherapy.

In the randomized phase Ⅲ NAPOLI‐1 trial, the median overall survival (OS) was 6.1 months (95% confidence interval [CI] 4.8–8.9) for the combination of nal‐IRI/5‐FU/LV, whereas it was 4.2 months (95% CI 3.3–5.3) for 5‐FU/LV alone, with a hazard ratio (HR) of 0.67 (95% CI 0.49–0.92; *p* = 0.012), in patients with metastatic pancreatic cancer after GEM‐based chemotherapy.^12^ Based on the findings from the NAPOLI‐1 trial, the Food and Drug Administration approved nal‐IRI/5‐FU/LV in 2015, and the NCCN guidelines recommend this regimen as category 1.^11^ However, the OS with nal‐IRI/5‐FU/LV^12^, ^13^, ^14^ was not significantly superior to that with previously reported 5‐FU‐based chemotherapy regimens. For example, OFF was superior to 5‐FU/LV, with a median OS of 5.9 months in the phase III CONKO‐003 trial,^15^ and mFOLFOX6 showed a median OS of 6.1 months in the phase III PANCREOX trial, although it did not meet the primary endpoint.^16^ Furthermore, FOLFOX had a similar treatment effect to that of nal‐IRI/5‐FU/LV in a meta‐analysis of 11 randomized controlled trials.^17^ Although the NCCN guidelines recommend multiple second‐line chemotherapy regimens for unresectable pancreatic cancer, including nal‐IRI/5‐FU/LV as category 1, the preferred regimen is described as none^11^; hence, it is not clear which is the most preferred second‐line chemotherapy regimen for unresectable pancreatic cancer.

Several studies have reported the clinical utility of mFOLFIRINOX as a second‐line treatment for patients with unresectable pancreatic cancer.^18^, ^19^, ^20^ mFOLFIRINOX has a better safety profile than FOLFIRINOX as first‐line therapy.^6^ However, there are concerns regarding its safety when used as a second‐line treatment because patient PS is generally lower at second‐line treatment initiation than that at first‐line treatment initiation. To address this concern, we retrospectively compared the efficacy and safety of mFOLFIRINOX with those of a preplanned sequential regimen of upfront FOLFIRI followed by a switch to FOLFOX after disease progression as a second‐line treatment regimen for patients with unresectable pancreatic cancer.

## 2 METHODS

### 2.1 Patients

This retrospective single‐center analysis was conducted at the Department of Hepatobiliary and Pancreatic Medical Oncology, Kanagawa Cancer Center, Yokohama.^21^ We included all patients who initiated treatment with mFOLFIRINOX or sequential chemotherapy as a second‐line treatment after GEM‐based chemotherapy for unresectable pancreatic cancer at our institution between December 2014 and May 2019. At our institution, as a second‐line treatment for unresectable pancreatic cancer with good PS (ECOG PS score of 0 or 1), mFOLFIRINOX was the first choice until July 2018, and thereafter sequential chemotherapy was the first choice. The sequential chemotherapy group included all patients who initiated sequential chemotherapy (FOLFIRI/FOLFOX), even if they completed the FOLFIRI regimen, irrespective of the completion of the subsequent FOLFOX regimen. Patients with histologically or cytologically confirmed pancreatic carcinoma previously treated with GEM‐based chemotherapy were eligible. Patients with unresectable pancreatic cancer with or without metastases were included in the study. All patients had an ECOG PS score of 0 or 1 and had adequate bone marrow and renal function. Patients whose starting dose of the drugs was reduced, including those with uridine diphosphate glucuronosyltransferase (UGT) genetic polymorphisms such as homozygous UGT1A1*28 or UGT1A1*6 and heterozygous UGT1A1*28 and UGT1A1*6UGT1A1, were excluded.

### 2.2 Treatment

The treatment sequences for chemotherapy in this study are shown in Figure S1. Patients were treated with mFOLFIRINOX or sequential chemotherapy (FOLFIRI/FOLFOX) every 2 weeks per cycle. Patients in the mFOLFIRINOX group were treated with a 2‐h intravenous infusion of oxaliplatin 85 mg/m^2^, a 2‐h intravenous infusion of LV 200 mg/m^2^, a 90‐min intravenous infusion of irinotecan 150 mg/m^2^, and a continuous 46‐h intravenous infusion of 5‐FU 2400 mg/m^2^ without bolus 5‐FU infusion.^6^ Patients in the sequential chemotherapy group were initially treated with FOLFIRI, administered as a 2‐h intravenous infusion of LV 200 mg/m^2^, a 90‐min intravenous infusion of irinotecan 150 mg/m^2^, and a continuous 46‐h intravenous infusion of 5‐FU 2400 mg/m^2^ without bolus 5‐FU infusion. At disease progression during FOLFIRI, with good PS, chemotherapy was switched to FOLFOX, administered as a 2‐h intravenous infusion of oxaliplatin 85 mg/m^2^, a 2‐h intravenous infusion of LV 200 mg/m^2^, and a continuous 46‐h intravenous infusion of 5‐FU 2400 mg/m^2^ without bolus 5‐FU infusion. All treatments were continued until disease progression, unacceptable toxicity, or patient refusal.

During treatment, the dose of the drugs was reduced according to the patient's condition, if needed. All patients routinely received palonosetron, dexamethasone, and aprepitant as antiemetic prophylaxis.

### 2.3 Efficacy and safety evaluation

We compared the efficacy and safety of mFOLFIRINOX and sequential chemotherapy. Tumor assessments were based on Response Evaluation Criteria in Solid Tumors version 1.1^22^ and were performed every 6–8 weeks. Efficacy was analyzed in patients with distant metastases or recurrence, excluding those with locally advanced pancreatic cancer (LAPC) (efficacy analysis population), and safety was analyzed in all patients, including those with LAPC (safety analysis population). For efficacy evaluation, the primary endpoint was OS, defined as the time from the date of treatment initiation to the date of death for any reason; patients who were alive were censored at the last follow‐up date. The secondary efficacy endpoints were as follows: (1) progression‐free survival (PFS), defined as the time from the date of treatment initiation to the date of documentation of disease progression or death for any reason; patients who were alive without progression during mFOLFIRINOX treatment or sequential chemotherapy were censored at the end of mFOLFIRINOX treatment or sequential chemotherapy; (2) objective response rate (ORR), define as the proportion of patients with the best overall response of unconfirmed complete response (CR) or partial response (PR); (3) and disease control rate (DCR), defined as the proportion of patients with the best overall response of unconfirmed CR, PR, or stable disease (SD). In the sequential chemotherapy group, the PFS in patients who could receive FOLFOX after FOLFIRI was defined as the time from the initiation of FOLFIRI to the date on which disease progression or death was confirmed due to any reason during FOLFOX treatment. The best overall response for the sequential chemotherapy group was defined as the best overall response for both prior FOLFIRI and subsequent FOLFOX. In contrast, the incidence of grade ≥3 adverse events (neutropenia, febrile neutropenia, anorexia, and diarrhea) was used as the endpoint for safety evaluation. Adverse effects were graded using the National Cancer Institute Common Terminology Criteria for Adverse Events version 5.0.^23^

### 2.4 Statistical analysis

OS and PFS were estimated using the Kaplan–Meier method. When comparing OS and PFS between the two groups, *p*‐value was calculated using the unstratified log‐rank test, and the HRs and 95% CIs were calculated using unstratified Cox regression. While analyzing the ORR and DCR, the odds ratio, 95% CI, and *p*‐value were calculated using a logistic regression model. Pretreatment data were collected for age, sex, ECOG PS, disease status (LAPC or not), metastatic sites (liver, lung, distant lymph node, and peritoneum), carcinoembryonic antigen (CEA), and carbohydrate antigen 19–9 (CA19‐9), and subgroup analysis was performed using non‐stratified Cox regression according to each factor. Quantitative data are expressed as medians (with ranges), and qualitative data are expressed as percentages. Continuous variables were dichotomized according to the median or reference value of each variable. Statistical analyses were performed using JMP PRO version 15.0.0 (SAS Institute Inc.). The clinical data cut‐off was September 19, 2019.

## 3 RESULTS

### 3.1 Patient characteristics

**FIGURE 1:**
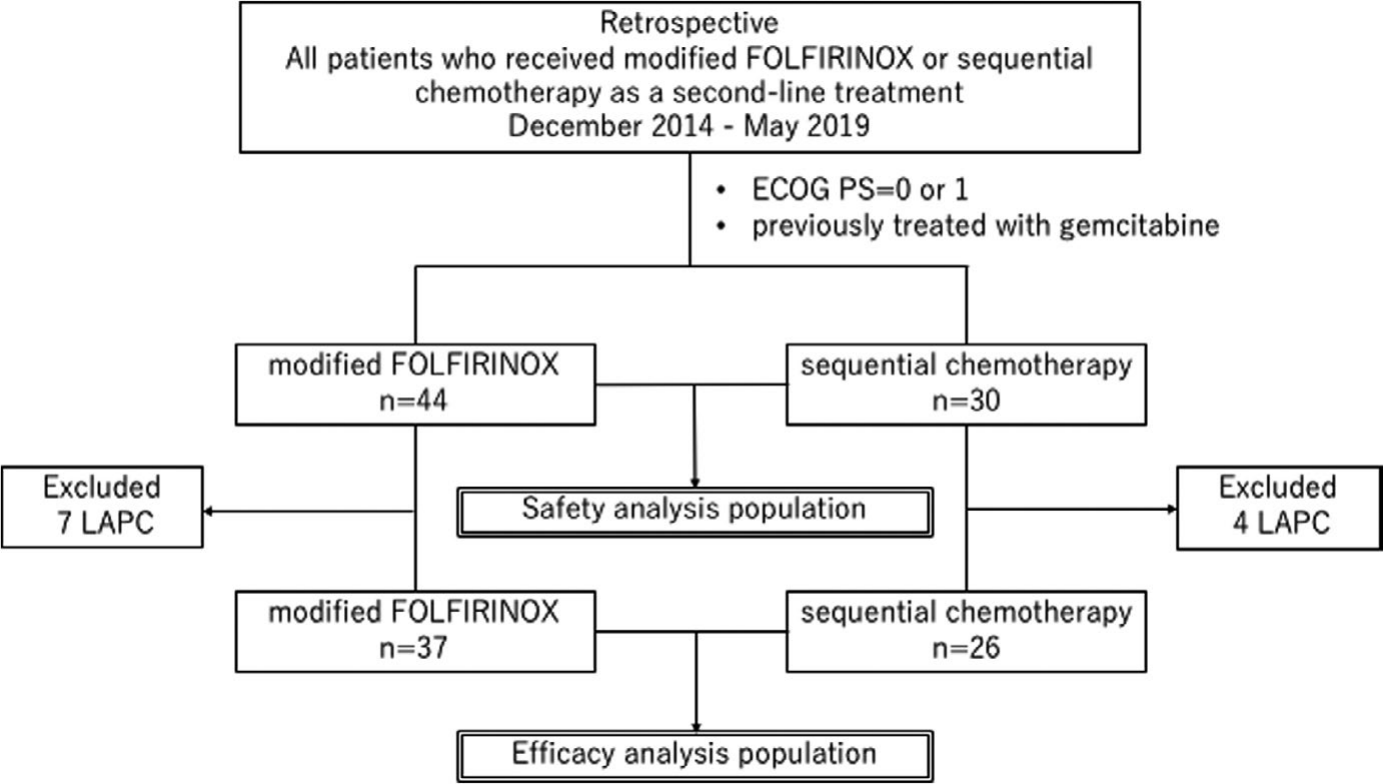
Patient selection flowchart. ECOG PS, Eastern Cooperative Oncology Group performance status; LAPC, locally advanced pancreatic cancer

A total of 74 patients treated at our institution between December 2014 and May 2019 were enrolled in this study and retrospectively examined. The mFOLFIRINOX group included 44 patients and the sequential chemotherapy group included 30 patients (safety analysis population) (Figure 1). The patient characteristics are shown in Table 1. There were no significant differences in the background characteristics of the groups. In the mFOLFIRINOX group, all patients received GnP, and in the sequential chemotherapy group, 28 patients received GnP, 1 patient received GEM plus S1, and 1 patient received GEM monotherapy.

**TABLE 1:** Characteristics of patients (safety analysis population)

	Modified FOLFIRINOX *n* = 44	Sequential chemotherapy *n* = 30
Age (years)		
Median (range)	64	67
Range	42–73	53–77
≥65 years (%)	20 (45.4)	22 (73.3)
≥75 years (%)	0	4 (13.3)
Sex		
Male (%)	23 (52.3)	19 (63.3)
Female (%)	21 (47.7)	11 (36.7)
ECOG PS		
0 (%)	22 (50.0)	16 (53.3)
1 (%)	22 (50.0)	14 (46.7)
LAPC (%)	7 (17.0)	4 (17.0)
Metastatic site		
Liver (%)	25 (56.8)	14 (46.6)
Lung (%)	11 (25.0)	7 (23.3)
Distant lymph node (%)	8 (18.2)	9 (30.0)
Peritoneum (%)	9 (20.5)	8 (26.7)
Bone (%)	3 (6.8)	2 (6.7)
CEA (ng/ml)		
Median	5.5	5.9
Range	0.9–1444.0	1.0–121.1
≤10 (%)	29 (65.9)	19 (63.3)
>10 (%)	15 (34.1)	11 (36.7)
CA19‐9 (IU/ml)		
Median	1489.1	483.9
Range	2.0–330 930	2.0–290 380
≤1000 (%)	22 (50.0)	20 (66.7)
>1000 (%)	22 (50.0)	10 (33.3)

Abbreviations: CA19‐9, carbohydrate antigen 19‐9; CEA, carcinoembryonic antigen; ECOG PS, Eastern Cooperative Oncology Group performance status; LAPC, locally advanced pancreatic cancer.

### 3.2 Treatment exposure

In the mFOLFIRINOX group, the median number of mFOLFIRINOX cycles per patient was 9 (range, 1–66). In contrast, in the sequential chemotherapy group, the median number of sequential chemotherapy (FOLFIRI/FOLFOX), preceding FOLFIRI, and subsequent FOLFOX cycles per patient was 5 (range, 1–51), 3 (range, 1–41), and 0 (range, 0–10), respectively. Among the 30 patients in the sequential chemotherapy group, 13 patients (12 patients in the efficacy analysis population) were able to receive both prior FOLFIRI and subsequent FOLFOX chemotherapy, and among them, progressive disease (PD) of prior FOLFIRI was determined by imaging in 7 patients and by elevated tumor markers, such as CEA and CA19‐9, in 6 patients. In the remaining 17 patients in the sequential chemotherapy group were unable to transfer to FOLFOX and ended with only prior FOLFIRI; among them, 6 patients were assessed to have PD with FOLFIRI, and PD of prior FOLFIRI was determined by imaging in 3 patients and by elevated tumor markers in 3 patients. The reasons for failure to switch to FOLFOX were poor general condition in 11 patients, request in 3 patients, and death in 1 patient; 2 patients were still receiving prior FOLFIRI at the time of data cut‐off.

### 3.3 Efficacy results

**FIGURE 2:**
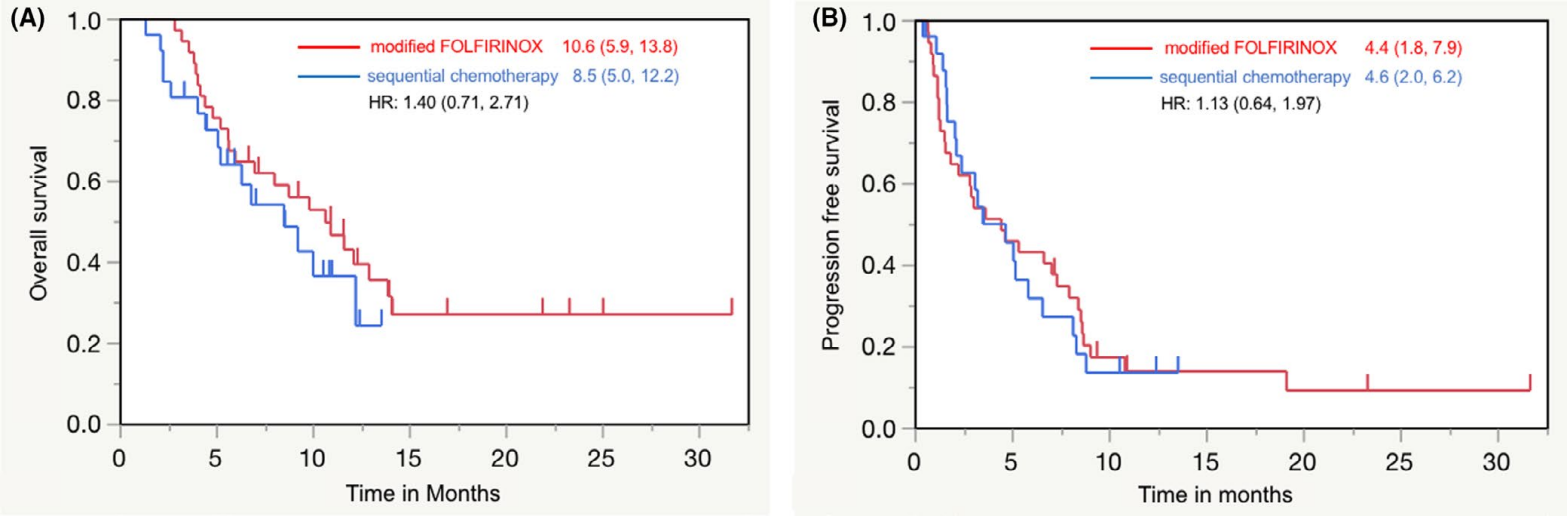
The Kaplan–Meier curves for (A) overall survival in the modified FOLFIRINOX group versus overall survival in the sequential chemotherapy group and (B) progression‐free survival in the modified FOLFIRINOX group versus overall survival in the sequential chemotherapy group (efficacy analysis population)

In the efficacy analysis population, the mFOLFIRINOX group included 37 patients and the sequential chemotherapy group included 26 patients. The primary efficacy endpoint of OS tended to be slightly prolonged in the mFOLFIRINOX group than that in the sequential chemotherapy group. The median OS was 10.6 months (95% CI: 5.9–13.8 months) in the mFOLFIRINOX group and 8.5 months (95% CI: 5.0–12.2 months) in the sequential chemotherapy group (unstratified HR [95% CI]: 1.40 [0.71–2.71]; *p* = 0.3177 [two‐sided *p*‐value]; Table 2; Figure 2). PFS, a secondary efficacy endpoint, was similar between the groups. The median PFS was 4.4 months (95% CI: 1.8–7.9 months) in the mFOLFIRINOX group and 4.6 months (95% CI: 2.0–6.2 months) in the sequential chemotherapy group (unstratified HR [95% CI]: 1.13 [0.64–1.97]; *p* = 0.6566 [two‐sided *p*‐value]; Table 2; Figure 2).

**TABLE 2:** Summary of efficacy results (efficacy analysis population)

	Modified FOLFIRINOX *n* = 37	Sequential chemotherapy *n* = 26	
Overall survival, months			
Median	10.6	8.5	
95% CI	5.9–13.8	5.0–12.2	
HR^a^	1.40		
95% CI	0.71–2.71		
*p*‐value^b^	0.3177		
Progression‐free survival, months			
Median	4.4	4.6	
95% CI	1.8–7.9	2.0–6.2	
HR^a^	1.13		
95% CI	0.64–1.97		
*p*‐value^b^	0.6566		
Best overall response, *n* (%)			
CR	0	0	
PR	3 (8.1)	1 (3.8)	
SD	21 (56.8)	10 (38.5)	
PD	13 (35.1)	12 (46.2)	
NE	0	3 (11.5)	
ORR (CR + PR), *n* (%)	3 (8.1)	1 (3.8)	
Odds ratio^c^	1.94		
95% CI	0.19–19.87		
*p*‐value^c^	1.0000		
DCR (CR + PR + SD), *n* (%)	24 (64.9)		11 (42.3)
Odds ratio^c^	3.68		
95% CI	1.23–10.99		
*p*‐value^c^	0.0303		

Abbreviations: CI, confidence interval; CR, complete response; DCR, disease control rate; HR, hazard ratio; NE, not evaluable; ORR, objective response rate; PD, progressive disease; PR, partial response; SD, stable disease.

^a^ Unstratified Cox proportional hazards modeling.

^b^ Two‐sided *p*‐value from the log‐rank test.

^c^ Logistic regression model.

**FIGURE 3:**
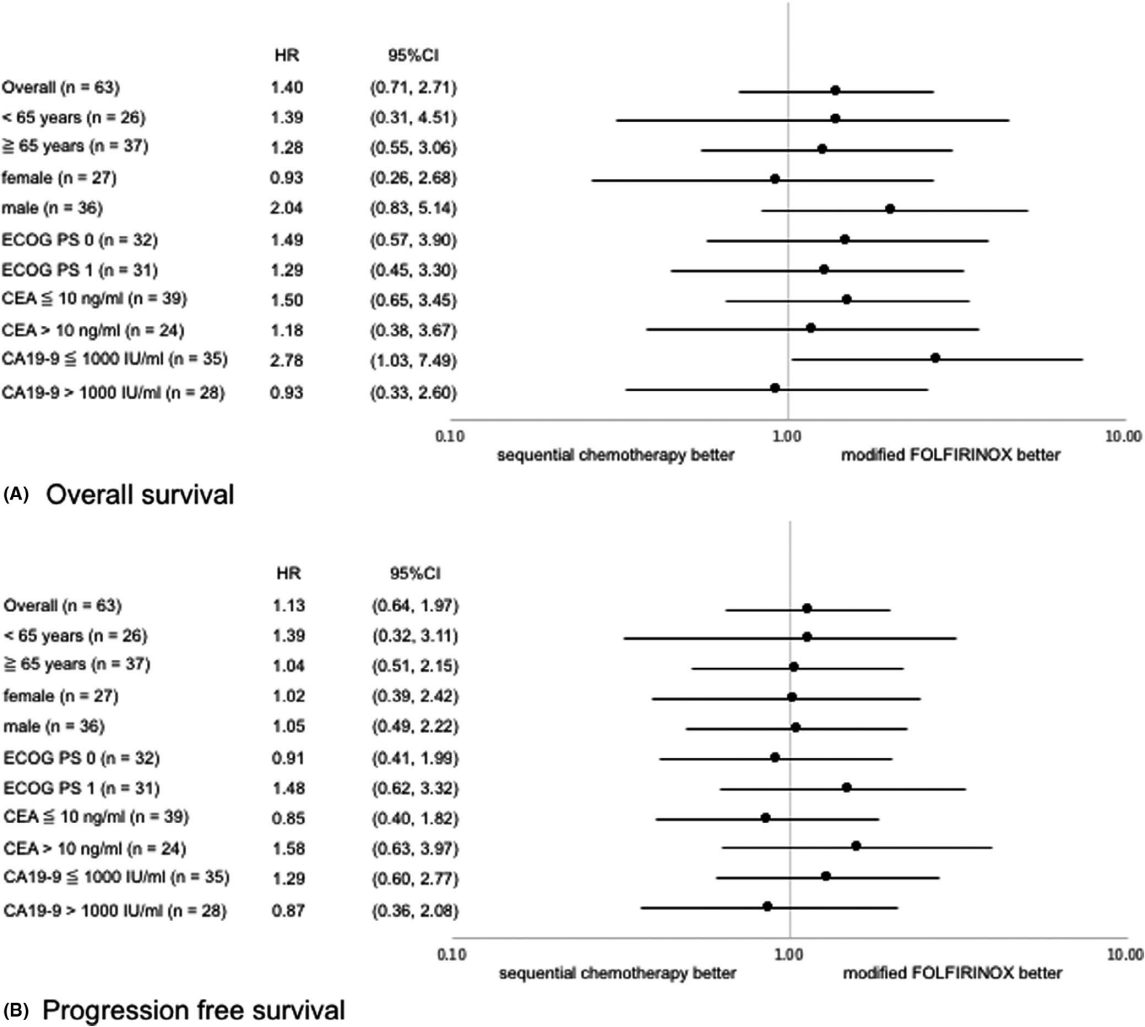
Forest plots of hazard ratios for (A) overall survival and (B) progression‐free survival (efficacy analysis population). CA19‐9, carbohydrate antigen 19‐9; CEA, carcinoembryonic antigen; ECOG PS, Eastern Cooperative Oncology Group performance status

Similar to OS, the ORR and especially DCR tended to be better in the mFOLFIRINOX group than in the sequential chemotherapy group. The best overall response in the mFOLFIRINOX group was PR (8.1%), SD (56.8%), and PD (35.1%). In contrast, the best overall response in the sequential chemotherapy group was PR (3.8%), SD (38.5%), and PD (46.2%). In the sequential chemotherapy group, prior to the first tumor assessment, treatment was discontinued in two patients and one patient due to patient request and adverse events, respectively, and the tumor assessment in three patients (11.5%) was not evaluable. The ORR was 8.1% (3/37 patients) in the mFOLFIRINOX group and 3.8% (1/26 patients) in the sequential chemotherapy group (odds ratio [95% CI]: 1.94 [0.19–19.87]; *p* = 1.0000 [two‐sided *p*‐value]; Table 2; Figure 3). The DCR was 64.9% (24/37 patients) in the mFOLFIRINOX group and 42.3% (11/26 patients) in the sequential chemotherapy group (odds ratio [95% CI]: 3.68 [1.23–10.99]; *p* = 0.0303 [two‐sided *p*‐value]; Table 2).

Among the 12 patients in the sequential chemotherapy group who were treated with subsequential FOLFOX, the best overall response for FOLFOX was SD in three (25.0%) patients and PD in nine (75.0%) patients. The median OS and PFS from the date of FOLFOX initiation in the 12 patients were 3.0 months (95% CI: 1.3–5.9 months) and 1.4 months (95% CI: 0.5–2.1 months), respectively.

Post‐treatment mFOLFIRINOX or FOLFIRI/FOLFOX was possible in 24.3% of (9/37) patients in the mFOLFIRINOX group and 15.4% of (4/26) patients in the sequential chemotherapy group. In the mFOLFIRINOX group, six patients received S‐1 monotherapy, two patients received GnP again, and one patient received S‐1 combination radiation therapy as a third‐line treatment. In the sequential chemotherapy group, three patients received S‐1 monotherapy and one patient received S‐1 combination radiation therapy as a third‐line treatment.

We performed exploratory subgroup analysis of OS and PFS (Figure 3A,B). In subgroup analysis of most factors, there were no significant differences in OS or PFS between the groups, but for CA19‐9 ≤1000 IU/ml, OS was significantly better in the mFOLFIRINOX group than in the sequential chemotherapy group (HR 2.78, 95% CI 1.03–7.49]).

### 3.4 Safety results

The results of the endpoint for safety evaluation, the incidence of grade ≥3 adverse events (neutropenia, febrile neutropenia, anorexia, and diarrhea), are shown in Table 3. Grade ≥3 rates of neutropenia, febrile neutropenia, and anorexia were 40.9%, 6.8%, and 18.2%, respectively, in the mFOLFIRINOX group and 3.3%, 0%, and 3.3%, respectively, in the sequential chemotherapy group. Only one (3.3%) patient in the sequential chemotherapy group discontinued treatment due to adverse events. The patient developed grade 3 diarrhea during the second cycle of FOLFIRI, which improved with treatment discontinuation, followed by treatment with S‐1 monotherapy as third‐line treatment. The adverse event was determined to be diarrhea due to plexus invasion of pancreatic body cancer, and its causal relationship to treatment was ruled out.

**TABLE 3:** Incidence of grade ≥3 adverse events (safety analysis population)

CTCAE ver. 5.0	Modified FOLFIRINOX *n* = 44	Sequential chemotherapy *n* = 30
Neutropenia (%)	18 (40.9)	1 (3.3)
Febrile neutropenia (%)	3 (6.8)	0
Anorexia (%)	8 (18.2)	1 (3.3)
Nausea (%)	7 (15.9)	0
Diarrhea (%)	2 (4.5)	1 (3.3)

Abbreviation: CTCAE, National Cancer Institute Common Terminology Criteria for Adverse Events.

## 4 DISCUSSION

We compared the efficacy and safety of mFOLFIRINOX and sequential chemotherapy as a second‐line treatment for unresectable pancreatic cancer. Although the difference between both groups was not significant, the Kaplan–Meier curves showed that OS, the primary endpoint, tended to be consistently higher in the mFOLFIRINOX group than in the sequential chemotherapy group, indicating a trend toward slightly better efficacy of mFOLFIRINOX. Regarding the secondary endpoints, ORR and especially DCR also tended to be better in the mFOLFIRINOX group than in the sequential chemotherapy group, similar to OS, but PFS was similar in both groups. The discrepancy between OS and PFS results could be influenced by two factors—unlike the mFOLFIRINOX group, the sequential chemotherapy group included patients aged ≥75 years (4/26 [15.4%] patients), and the sequential chemotherapy group may have had a worse prognosis; the sequential chemotherapy group had a lower rate of post‐treatment than the mFOLFIRINOX group (15.4% vs. 24.3%). In contrast to efficacy results, safety results showed that the incidence of grade ≥3 neutropenia, febrile neutropenia, and anorexia was significantly lower in the sequential chemotherapy group than in the mFOLFIRINOX group, indicating that the safety of sequential chemotherapy was better.

As a second‐line treatment for unresectable pancreatic cancer, the clinical utility of nal‐IRI/5‐FU/LV has been demonstrated in the phase III NAPOLI‐1 trial.^12^, ^14^ However, the NCCN guidelines recommend multiple chemotherapy regimens, including nal‐IRI/5‐FU/LV as category 1, followed by FOLFIRINOX, mFOLFIRINOX, FOLFIRI, OFF, and FOLFOX, as a second‐line treatment after GEM‐based chemotherapy, and the preferred regimen is described as none.^11^ Although the regulatory approval of nal‐IRI/5‐FU/LV has clarified the principle of treatment, the selection of second‐line chemotherapy regimen for unresectable pancreatic cancer is controversial. In such circumstances, several studies have confirmed the efficacy and safety of mFOLFIRINOX as a second‐line treatment; its clinical usefulness as a first‐line treatment has already been established.^6^, ^18^, ^19^, ^24^ The consideration of mFOLFIRINOX, the standard first‐line treatment alongside GnP, as a second‐line treatment is natural and reasonable in cases where it has not been used as a first‐line treatment. However, even with a modified regimen, FOLFIRINOX, which is a combination of the three cytotoxic agents, is associated with adverse reactions such as myelosuppression.^6^ The safety of mFOLFIRINOX as a second‐line treatment is a concern, given that patient PS at second‐line treatment initiation is generally worse than that at first‐line treatment initiation. Therefore, in this study, we investigated the clinical utility of sequential chemotherapy initiated with FOLFIRI followed by FOLFOX. Peripheral neuropathy is a typical side effect of GnP, a standard first‐line therapy.^25^, ^26^, ^27^ In patients with peripheral neuropathy who are transferred to second‐line mFOLFIRINOX, the added oxaliplatin may exacerbate peripheral neuropathy.^28^ Although peripheral neuropathy rarely becomes serious if appropriately managed with drug interruption or dose reduction, there are concerns regarding reduced quality of life. Therefore, in this study, we developed a strategy for sequential chemotherapy, in which oxaliplatin‐free FOLFIRI was used first instead of oxaliplatin‐containing regimens for second‐line treatment.

In the present study, slight tendency of prolongation of OS, the primary endpoint, was observed in the mFOLFIRINOX group, however, the difference was not significant. Given the better safety observed in the sequential chemotherapy group, the risk–benefit balance of sequential chemotherapy was better than that of mFOLFIRINOX. Considering that patient PS is generally lower at second‐line treatment initiation with disease progression than that at first‐line treatment initiation, safety appears to be more important in the selection of second‐line chemotherapy regimen. Therefore, sequential chemotherapy with better risk–benefit balance can be considered a second‐line treatment option for patients with unresectable pancreatic cancer. Among the regimens recommended as a second‐line treatment for unresectable pancreatic cancer in the NCCN guidelines, nal‐IRI/5‐FU/LV is recommended as category 1.^11^ The median OS with nal‐IRI/5‐FU/LV in the NAPOLI‐1 trial and the Japanese randomized controlled phase II study was 6.1 and 6.3 months, respectively.^12^, ^13^ Although the comparison between different studies and evaluation are limited, the median OS of 8.5 months with sequential chemotherapy in our study was not inferior to that with nal‐IRI/5‐FU/LV, and clinical utility can be expected.

Although sequential chemotherapy can be considered a second‐line treatment option with better favorable risk–benefit balance than mFOLFIRINOX, mFOLFIRINOX cannot be ruled out as a second‐line treatment option, given the slightly prolonged tendency of OS, as well as the higher DCR and the absence of adverse events leading to treatment discontinuation in the present study. In the NCCN guidelines, mFOLFIRINOX, used as control treatment in this study, is recommended in category 2A as a second‐line treatment for unresectable pancreatic cancer.^11^ In the phase III ACCORD11 trial, the efficacy of FOLFIRINOX as a first‐line treatment was superior to that of GEM,^5^ although the trial revealed several safety concerns, such as febrile neutropenia and anorexia. The efficacy and safety of mFOLFIRINOX as a first‐line treatment have been demonstrated^6^, ^24^ and those of mFOLFIRINOX as a second‐line treatment have been reported; the reported median OS with mFOLFIRINOX as a second‐line treatment ranges from 7.0 to 10.3 months.^18^, ^19^, ^20^ In our study, the median OS of 10.6 months in the mFOLFIRINOX group was not inferior to that reported in previous studies, although this is a comparison between different trials and requires careful consideration. Although the safety results of the mFOLFIRINOX group in this study were inferior to those of the sequential chemotherapy group, there were no clear differences from the results of previous studies on mFOLFIRINOX, and the results were tolerable.^6^, ^18^, ^19^, ^20^, ^24^

We also performed exploratory subgroup analysis to investigate appropriate patient selection for mFOLFIRINOX and sequential chemotherapy; however, the results were not meaningful because of the small number of cases and events in the subgroups. In this study, the risk–benefit balance of sequential chemotherapy can be considered more favorable than that of mFOLFIRINOX, providing support for the consideration of the selection of sequential chemotherapy as a second‐line treatment, especially in patients with residual peripheral neuropathy and in those with concerns regarding the ECOG PS after first‐line GnP treatment.

The three main limitations of the present study were the single‐center retrospective study design in Japan, small sample size that contributed to the lack of power, and the lack of biomarker assays such as *BRCA* mutation analysis. In the future, for patients with *BRCA* mutations who may benefit from platinum‐based anticancer agents, FOLFOX should precede FOLFIRI as sequential chemotherapy.^29^

## 5 CONCLUSIONS

Given the tendency toward slightly longer OS and higher DCR with mFOLFIRINOX than with sequential chemotherapy in this study, mFOLFIRINOX may be considered a second‐line treatment option for unresectable pancreatic cancer. However, given the higher incidence of grade ≥3 adverse events with mFOLFIRINOX than with sequential chemotherapy in this study, sequential chemotherapy is a regimen with better favorable risk–benefit balance than mFOLFIRINOX, and can be considered a second‐line treatment option for patients with unresectable pancreatic cancer. Prospective clinical studies with larger sample sizes are warranted to confirm the results of this study.

## CONFLICT OF INTEREST

The authors declare that they have no known competing financial interests, personal relationships, or competing interests that could have influenced the work reported in this paper.

## AUTHORS’ CONTRIBUTIONS

Shun Tezuka and Makoto Ueno designed the study. Shun Tezuka acquired and analyzed the patient data. Shun Tezuka and Makoto Ueno participated in the interpretation of results. Shun Tezuka drafted the manuscript. All authors have read, reviewed, and approved the final manuscript.

## ETHICAL APPROVAL STATEMENT

This study was approved by the Institutional Review Board of Kanagawa Cancer Center (approval number: 2021‐20). The need for informed consent was waived owing to the retrospective nature of the study. This study has been listed on the Kanagawa Cancer Center website (http://kcch.kanagawa‐pho.jp/general/cr‐shoukakinaika‐kantansui.html).

## Supporting information

Figure S1

## Data Availability

The data that support the findings of this study are available from the corresponding author upon reasonable request.
