# Supplementary figures and images for: Modified FOLFIRINOX versus sequential chemotherapy (FOLFIRI/FOLFOX) as a second‐line treatment regimen for unresectable pancreatic cancer: A real‐world analysis
Source: Cancer Med. 2021 Dec 24;11(4):1088–98. doi: 10.1002/cam4.4512 (PMC8855892; doi:10.1002/cam4.4512)

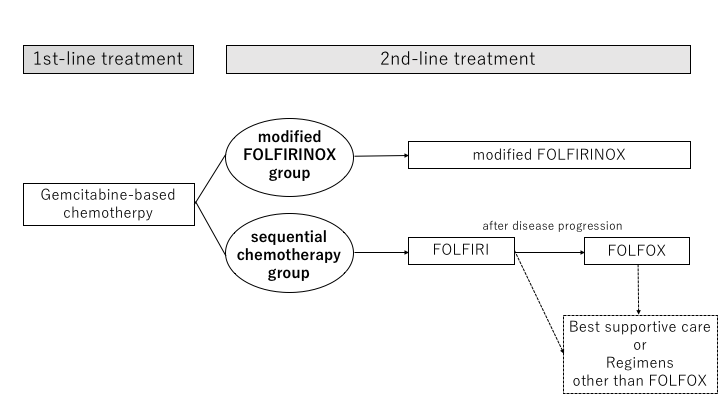

Supplement: Supplementary file 1 — Figure S1 [file CAM4-11-1088-s001.tiff]
